# Characteristics and Outcomes of Traumatic Acute Subdural Hematoma in Elderly Patients Receiving Antithrombotic Therapy: A Single-Center Retrospective Cohort Study

**DOI:** 10.3390/jcm15062163

**Published:** 2026-03-12

**Authors:** Tsuyoshi Ohta, Masaomi Koyanagi, Masanori Goto, Tadashi Sunohara, Nobuyuki Fukui, Tomomi Ishikawa, Yasuhiro Yamamoto, Mikako Nomoto, Takateru Takamatsu, Masanori Tokuda, Hikari Tomita, Mai Yoshimoto, Takenori Ohga, Nobuyuki Sakai

**Affiliations:** Department of Neurosurgery, Kobe City Medical Center General Hospital, Kobe 650-0047, Japan; masaomi_koyanagi@kcho.jp (M.K.); masanori_goto@kcho.jp (M.G.); tadashi_sunohara@kcho.jp (T.S.); nobuyuki_fukui@kcho.jp (N.F.); tomomi_ishikawa@kcho.jp (T.I.); yasuhiro_yamamoto@kcho.jp (Y.Y.); mikako_nomoto@kcho.jp (M.N.); takateru_takamatsu@kcho.jp (T.T.); masanori_tokuda@kcho.jp (M.T.); hikari_tomita@kcho.jp (H.T.); mai_yoshimoto@kcho.jp (M.Y.); takenori_oga@kcho.jp (T.O.); nosakai@shimizu-hospital.or.jp (N.S.)

**Keywords:** acute subdural hematoma, antithrombotic therapy, elderly

## Abstract

**Background:** Antithrombotic therapy is a risk factor for subdural hematoma after head injury. **Methods:** We retrospectively studied 180 consecutive patients with traumatic acute subdural hematoma. **Results**: The median age was 81 years, 68 (38%) were female, and 44% were on antithrombotic therapy. In the antithrombotic therapy group, the patients were significantly older (84, 77–88 vs. 78, 74–84, *p* value = 0.00104), and the proportion of minor injury was significantly higher (83% vs. 61%, *p* value = 0.00178). Poor clinical outcomes were not significantly different between the groups (44% vs. 41%; *p* value = 0.762). In multivariable logistic regression analysis adjusted for age and sex, poor outcomes (42%) were associated with the first Glasgow coma scale scores (OR 0.73, 95% CI 0.65–0.82, *p* value < 0.001) and the first CT findings (OR 4.9, 95% CI 1.98–11.8, *p* value < 0.001), but not with antithrombotic therapy (OR 1.48, 95% CI 0.61–3.60, *p* value = 0.390). Of the 97 patients treated conservatively for more than 2 weeks, surgical intervention in the chronic phase was higher in the antithrombotic therapy group (16% vs. 1.9%; *p* value = 0.0230). The timing of re-administration did not correlate with the incidence of chronic surgical intervention (within 2 weeks: 18%; over 2 weeks: 9.1%; *p* value = 0.663). **Conclusions:** The proportion of minor injury was significantly higher in the antithrombotic therapy group than in the non-medication group. Antithrombotic therapy was not associated with poor outcome but correlated with the increased risk of surgical intervention in the chronic phase.

## 1. Introduction

With the recent proliferation of medical and endovascular treatment for ischemic stroke and cardiovascular disease, the number of individuals receiving antithrombotic therapy has markedly increased in routine clinical practice. For example, among patients aged 80 years and older with atrial fibrillation, the rate of anticoagulant prescriptions rose fivefold—from approximately 10% in 2001 to 46% in 2015 [1]. This dramatic rise in anticoagulant use among the elderly reflects evolving guidelines and growing recognition of stroke prevention in atrial fibrillation.

The management of antiplatelet and antithrombotic agents before, during, and after endovascular neurointerventional procedures is recommended for select patient populations. Such peri-procedural pharmacologic strategies are particularly advised for individuals undergoing neurointerventional treatment for brain aneurysms or carotid artery stenting [2].

Antithrombotic drugs have been identified as a contributing factor in the development of subdural hematoma (SDH) [3]. These agents are recognized as potential risks for intracranial bleeding, especially in the context of trauma or underlying vascular fragility. Traumatic injury to the head is the most frequent precipitating event for the onset of acute SDH.

Acute SDH is observed in approximately 11% of mild to moderate head injuries requiring hospitalization, and in about 20% of severe traumatic brain injuries [4]. The prevalence of acute SDH rises with injury severity, making it a common finding in hospitalized head trauma cases. Acute SDH tends to follow a more severe clinical course [5], particularly among elderly patients [6].

However, the specific characteristics of acute SDH in patients receiving antithrombotic therapy, as well as the optimal timing for resuming such medications, remain poorly defined. There is a lack of consensus and clarity regarding how antithrombotic use influences the presentation and management of acute SDH.

The purpose of this study was to elucidate the clinical features of acute SDH in elderly patients receiving antithrombotic therapy and to provide detailed insights into how antithrombotic therapy affects the course and management of acute SDH in the aging population.

## 2. Materials and Methods

This study was designed as a single-center retrospective cohort investigation. The study population consisted of 180 consecutive patients who presented with traumatic acute subdural hematoma (SDH) as their primary diagnosis. These individuals were all first-time visitors to the hospital and were admitted and treated between October 2017 and April 2022, ensuring a continuous and unbroken dataset without selective case inclusion. Clinical information for each patient—including demographics, comorbidities, medication history, neurological status, and laboratory values—was obtained from electronic medical records. In addition, all relevant imaging data were reviewed to ensure accurate classification of the hematoma and associated injuries. The diagnosis of acute SDH was established through neuroimaging, most commonly noncontrast head computed tomography (CT). In this study, CT was routinely performed as part of the initial evaluation for any patient with a history of head injury, regardless of the severity of symptoms, ensuring that even subtle cases were identified. For the purposes of this study, acute SDH was defined as a hematoma identified in patients who presented to the hospital within 1 to 2 days after symptom onset or traumatic impact. This time-based definition distinguishes acute SDH from subacute or chronic forms, which have different pathophysiological mechanisms and clinical courses.

Because the majority of patients were elderly and therefore less able to tolerate aggressive or highly invasive interventions, the institution followed a set of pragmatic treatment principles during the study period.

1. Medical management was prioritized whenever feasible, focusing on close monitoring, blood pressure control, reversal of coagulopathy when appropriate, and supportive care.
2. When surgical intervention was deemed necessary, it was performed as early as possible to prevent neurological deterioration, recognizing that timely decompression can be critical in acute SDH.
3. Minimally invasive procedures, such as burr-hole trepanation and hematoma irrigation, were preferred—particularly in the chronic phase—because they impose less physiological stress on frail, elderly patients and are associated with fewer perioperative complications.

The following clinical and radiological variables were examined to characterize the cohort and identify factors associated with outcomes: basic patient demographics such as age and sex, the Glasgow Coma Scale (GCS) score at the time of admission, the mechanism of injury leading to the acute subdural hematoma, the presence or absence of antithrombotic therapy, imaging findings on the initial head CT scan, the Glasgow Outcome Scale (GOS) score at discharge or at the 3-month follow-up, and whether surgical intervention was performed.

Patients were considered to be receiving antithrombotic therapy if they had been taking any antiplatelet agent—including aspirin, clopidogrel, or cilostazol—or any anticoagulant medication, such as warfarin or direct oral anticoagulants (dabigatran, rivaroxaban, apixaban, or edoxaban), for at least 14 days prior to the index traumatic event. This definition ensured that only patients with stable, ongoing antithrombotic exposure were included, thereby excluding those who may have taken these medications sporadically or only briefly, which could confound the interpretation of bleeding risk.

Imaging findings on the first head CT were systematically evaluated for two key markers of severity: the presence of a midline shift greater than 5 mm [4], which reflects significant mass effect and intracranial pressure elevation, and the presence of concurrent intraparenchymal hemorrhage [7], which indicates more extensive traumatic brain injury and is known to worsen prognosis.

A poor clinical outcome was defined as a Glasgow Outcome Scale score of 1 or 2 at discharge or at the 3-month evaluation. This threshold corresponds to death (GOS 1) or a persistent vegetative state (GOS 2), aligning with commonly used outcome definitions in traumatic brain injury research.

Surgical interventions were categorized into two clinically meaningful groups: emergency operations, defined as procedures performed within the first 24 h after onset, and chronic-phase surgeries, defined as operations conducted more than 14 days after the initial injury.

Because the majority of traumatic SDH cases arise from motor vehicle accidents, falls, and assaults [4], the mechanisms of injury were simplified into two categories to facilitate analysis: major injury, which included high-energy events such as traffic accidents or significant falls, and minor injury, which encompassed lower-energy mechanisms such as pedestrian collisions, ground-level falls with bruising, or other relatively mild traumatic events.

Data was analyzed anonymously using retrospectively collected information. Dichotomous categorical data, such as sex and medication, were analyzed using Fisher’s exact test. The Mann–Whitney U test was employed to analyze ordinal or nonparametric, continuous data such as age and GCS. Noncategorical data are presented as the median and the interquartile range (25–75%). Explanatory variables for multivariable logistic regression models were restricted to ≈approximately 10% of patients in the less-frequent category [8]. All multivariable logistic regression analyses were conducted with age and sex adjustments. Odds ratios and 95% confidence intervals were estimated for variables with significant associations. The statistical significance level was set at *p* value < 0.05. All statistical analyses were conducted using EZR version 1.55 (Saitama Medical Center, Jichi Medical University, Saitama, Japan [9]).

## 3. Results

### 3.1. Patient Characteristics and Clinical Outcomes

The median age was 81 years (first and third quartiles 75–86), 68 (38%) were female, and 80 (44%) were on some form of antithrombotic therapy (31 on single antiplatelet agents, 8 on dual agents, 31 on anticoagulants, and 10 on both antiplatelet and coagulant agents). Details of the distributions and indications of antithrombotic therapy are shown in Table 1. No patients received anticoagulant reversal or platelet infusion.

As shown in Table 2, surgical interventions were performed in 63 patients (35%). 55 received emergency operations, consisting of 34 craniectomies, 20 craniotomies, and 1 trepanation with irrigation. In the antithrombotic therapy group, the patients were significantly older (84, 77–88 vs. 78, 74–84, *p* value = 0.00104), and the proportion of minor injury was significantly higher in comparison to the non-antithrombotic group (83% vs. 61%, *p* value = 0.00178). However, poor clinical outcomes were not significantly different between the groups (44% vs. 41%; *p* value = 0.762) (Table 2).

As shown in Table 3, in multivariable logistic regression analysis adjusted for age and sex, Glasgow Outcome Scale 1 or 2 at discharge or 3 months (76 cases, 42%) was associated with the Glasgow coma scale scores on admission (Odds ratio 0.73, 95% CI 0.65–0.82, *p* value < 0.001) and the first CT findings of midline shift of greater than 5 mm or concurrent brain contusion (Odds ratio 4.9, 95% confidence interval 1.98–11.8, *p* value < 0.001), but not with antithrombotic therapy (Odds ratio 1.48, 95% confidence interval 0.61–3.60, *p* value = 0.390) (Table 3).

### 3.2. Chronic Phase Surgical Interventions

Of 97 patients treated conservatively for more than 2 weeks, surgical interventions in the chronic phase were performed in 8 cases, consisting of 2 craniectomies, 4 craniotomies, and 3 trepanations with irrigation. The proportion of interventions was significantly higher among those who received antithrombotic therapy (7 of 45 cases, 16% vs. 1 of 52 cases, 1.9%; *p* value = 0.0230). The timing of resumption of antithrombotic therapy did not correlate with the incidence of chronic surgical intervention (within 2 weeks: 6 of 34, 18%; beyond 2 weeks: 1 of 11, 9.1%; *p* value = 0.663).

## 4. Discussion

This study revealed that antithrombotic therapy was associated with the occurrence of acute SDH following minor injury, and more cases required surgical intervention during the chronic phase, although it was not associated with a rise in poor outcomes. In other words, while antithrombotic therapy was associated with a higher risk of hematoma formation and subsequent surgery, it was not associated with the overall clinical prognosis.

Risk factors for a poor outcome in acute SDH include older age [10] and a lower GCS score [11]. As for imaging findings, head CT findings, such as a midline shift greater than 5 mm and acute traumatic intraparenchymal hemorrhage [12,13], correlate with poor outcome after acute SDH.

Antiplatelet and anticoagulant agents are used to prevent and manage thromboembolic disease in patients with a history of or at risk of cerebrovascular events, acute coronary syndromes, percutaneous coronary or vascular interventions with stenting, or peripheral arterial disease. Such medications are essential in maintaining vascular patency and reducing ischemic complications in high-risk populations. Patients are at high risk of complications such as stent thrombosis if antiplatelet therapy is discontinued prematurely [14]. Early cessation of these agents can jeopardize vascular integrity and lead to life-threatening thrombotic events.

In managing trauma patients receiving antithrombotic therapy, physicians often encounter a dilemma regarding whether to discontinue the medication. This therapeutic conflict complicates clinical decision-making, as both bleeding and thrombotic risks must be weighed carefully. Clinicians must balance the competing hazards of hemorrhage and ischemia when planning interventions. Our study found that discontinuing antithrombotic therapy for more than two weeks did not correlate with a lower rate of surgical intervention in the chronic phase. Resuming antithrombotic therapy shortly after achieving hemostasis may be a reasonable strategy to prevent ischemic complications without exacerbating chronic hematoma expansion. Early reinitiation of treatment could offer vascular protection while minimizing the risk of hematoma progression. However, this study included only retrospective data from a single center and did not systematically analyze various ischemic endpoints, so conclusions regarding thromboembolic events after discontinuation or resumption of antithrombotic therapy should be interpreted with caution.

We mainly performed craniotomies and craniectomies as emergency surgical interventions for acute SDH in this study group. However, various reports suggested that endoscopic hematoma evacuation was safe and effective for acute and subacute SDH compared with craniotomy in elderly patients [15,16,17]. Deploying endoscopic evacuation could be better for carefully selected cases.

Our study has several limitations. First, due to its retrospective design, treatment decisions were often influenced by patient-specific factors, and the outcomes should be interpreted with caution due to potential bias. Those potential biasing factors include age, frailty, comorbidities, injury characteristics, details of antithrombotic therapy, such as indication or intensity, and treatment-limitation decisions. Second, we observed that minor injuries in patients receiving antithrombotic therapy were significantly more associated with acute subdural hematoma. Nonetheless, because the cause of unidentified injuries is often unclear—especially in elderly patients—our findings may still be applicable to broader clinical contexts. Third, secondary outcomes—such as minor injury and chronic-phase surgery—were evaluated only by univariable analyses; due to the very small number of events, multivariable adjustment was not feasible, and these findings should be considered exploratory. Fourth, antithrombotic therapy was analyzed as a single composite exposure despite substantial heterogeneity, which may have obscured differential effects. Fifth, the analysis of antithrombotic resumption timing was underpowered and did not include ischemic endpoints; the absence of statistical significance should be interpreted as no detectable difference rather than no effect. Finally, our study did not include the patients treated with standardized reversal protocols such as Andexanet alfa because it was unavailable during the study period. As such, our data do not reflect outcomes associated with this reversal agent. However, our findings may help guide the selection of candidates for Andexanet alfa [18]. In light of an aging population and the growing adoption of advanced care planning, many trauma patients may opt to withdraw life-sustaining treatments following severe injury [19].

## 5. Conclusions

In this retrospective single-center study, the proportion of minor injury was significantly higher in the antithrombotic therapy group than in the non-medication group. Antithrombotic therapy was not associated with poor outcome but correlated with the increased risk of surgical intervention in the chronic phase. Discontinuation of antithrombotic therapy for more than 2 weeks did not reduce the incidence of surgical intervention in the chronic phase.

## Figures and Tables

**Table 1:** Distributions and indications of antithrombotic therapy.

Factors	Antiplatelet (*n* = 39)		Anticoagulant (*n* = 31)		Combination (*n* = 10)
	Single (*n* = 31)	Dual (*n* = 8)	Warfarin (*n* = 15)	Direct Oral Anticoagulant (*n* = 16)	
Cerebral infarction, *n*	10	2	5	1	2
Atrial fibrillation, *n*	1	0	4	11	3
Ischemic heart disease, *n*	8	2	1	1	3
Heart valve disease, *n*	3	0	4	0	2
Deep venous thrombosis, *n*	0	0	0	1	0
Others, *n*	1 lower extremity ischemia, 1 post-neuro intervention, and 1 coagulopathy	2 lower extremity ischemia and 1 post-neuro intervention	0	0	0
Unidentified, *n*	6	1	1	2	0

**Table 2:** Patient characteristics and clinical outcome.

Factors	Antithrombotic Therapy (*n* = 80)	No antithrombotic Therapy (*n* = 100)	*p* Value
Age	84 (77–88)	78 (74–84)	0.00104
Sex (female), *n* (%)	25 (31)	43 (43)	0.123
Glasgow Coma Scale on Admission	14 (6–15)	14 (6–15)	0.467
First CT findings of significant midline shift or concurrent brain contusion, *n* (%)	36 (45)	46 (46)	>0.999
Minor injury, *n* (%)	66 (83)	61 (61)	0.00178
Surgical interventions, *n* (%)	26 (33)	37 (37)	0.637
Poor outcome, *n* (%)	35 (44)	41 (41)	0.762

**Table 3:** Multivariable logistic regression analysis of Poor clinical outcome.

Factors	Odds Ratio	95% Confidence Interval	*p* Value
Age	0.99	0.93–1.04	0.606
Sex (female)	0.62	0.24–1.61	0.323
Glasgow Coma Scale	0.73	0.65–0.82	<0.001
First CT findings of significant midline shift or concurrent brain contusion	4.85	1.98–11.8	<0.001
Antithrombotic therapy	1.48	0.61–3.60	0.390

## Data Availability

The data supporting the results of this investigation are available from the first author upon reasonable request.

## Abbreviations

The following abbreviations are used in this manuscript:

SDH	Subdural hematoma
GCS	Glasgow Coma Scale

## References

[1] Dregan A., Ravindrarajah R., Charlton J., Ashworth M., Molokhia M. (2018). Long-term trends in antithrombotic drug prescriptions among adults aged 80 years and over from primary care: A temporal trends analysis using electronic health records. Ann. Epidemiol..

[2] Schirmer C.M., Bulsara K.R., Al-Mufti F., Haranhalli N., Thibault L., Hetts S.W. (2023). Antiplatelets and antithrombotics in neurointerventional procedures: Guideline update. J. NeuroInterv. Surg..

[3] Gaist D., Garcia Rodriguez L.A., Hellfritzsch M., Poulsen F.R., Halle B., Hallas J., Pottegård A. (2017). Association of Antithrombotic Drug Use with Subdural Hematoma Risk. JAMA.

[4] Bullock M.R., Chesnut R., Ghajar J., Gordon D., Hartl R., Newell D.W., Servadei F., Walters B.C., Wilberger J.E. (2006). Surgical management of acute subdural hematomas. Neurosurgery.

[5] Lee J.J., Segar D.J., Morrison J.F., Mangham W.M., Lee S., Asaad W.F. (2018). Subdural hematoma as a major determinant of short-term outcomes in traumatic brain injury. J. Neurosurg..

[6] Trevisi G., Sturiale C.L., Scerrati A., Rustemi O., Ricciardi L., Raneri F., Tomatis A., Piazza A., Auricchio A.M., Stifano V. (2020). Acute subdural hematoma in the elderly: Outcome analysis in a retrospective multicentric series of 213 patients. Neurosurg. Focus.

[7] Chang E.F., Meeker M., Holland M.C. (2007). Acute traumatic intraparenchymal hemorrhage: Risk factors for progression in the early post-injury period. Neurosurgery.

[8] Harrell F.E. (2015). Regression Modeling Strategies: With Applications to Linear Models, Logistic and Ordinal Regression, and Survival Analysis.

[9] Kanda Y. (2013). Investigation of the freely available easy-to-use software ‘EZR’ for medical statistics. Bone Marrow Transplant..

[10] Lukasiewicz A.M., Grant R.A., Basques B.A., Webb M.L., Samuel A.M., Grauer J.N. (2016). Patient factors associated with 30-day morbidity, mortality, and length of stay after surgery for subdural hematoma: A study of the American College of Surgeons National Surgical Quality Improvement Program. J. Neurosurg..

[11] Hatashita S., Koga N., Hosaka Y., Takagi S. (1993). Acute subdural hematoma: Severity of injury, surgical intervention, and mortality. Neurol. Med.-Chir..

[12] Koc R.K., Akdemir H., Oktem I.S., Menkü A. (1997). Acute subdural hematoma: Outcome and outcome prediction. Neurosurg. Rev..

[13] Hlatky R., Valadka A.B., Goodman J.C., Robertson C.S. (2004). Evolution of brain tissue injury after evacuation of acute traumatic subdural hematomas. Neurosurgery.

[14] Mathews R., Wang W., Kaltenbach L.A., Thomas L., Shah R.U., Ali M., Peterson E.D., Wang T.Y. (2018). Hospital Variation in Adherence Rates to Secondary Prevention Medications and the Implications on Quality. Circulation.

[15] Katsuki M., Kakizawa Y., Nishikawa A., Kunitoki K., Yamamoto Y., Wada N., Uchiyama T. (2020). Fifteen Cases of Endoscopic Treatment of Acute Subdural Hematoma with Small Craniotomy under Local Anesthesia: Endoscopic Hematoma Removal Reduces the Intraoperative Bleeding Amount and the Operative Time Compared with Craniotomy in Patients Aged 70 or Older. Neurol. Med.-Chir..

[16] Miki K., Nonaka M., Kobayashi H., Horio Y., Abe H., Morishita T., Iwaasa M., Inoue T. (2021). Optimal surgical indications of endoscopic surgery for traumatic acute subdural hematoma in elderly patients based on a single-institution experience. Neurosurg. Rev..

[17] Yokosuka K., Uno M., Matsumura K., Takai H., Hagino H., Matsushita N., Toi H., Matsubara S. (2015). Endoscopic hematoma evacuation for acute and subacute subdural hematoma in elderly patients. J. Neurosurg..

[18] Connolly S.J., Sharma M., Cohen A.T., Demchuk A.M., Członkowska A., Lindgren A.G., Molina C.A., Bereczki D., Toni D., Seiffge D.J. (2024). Andexanet for Factor Xa Inhibitor-Associated Acute Intracerebral Hemorrhage. N. Engl. J. Med..

[19] Leonard J.M., Polites S.F., Martin N.D., Glasgow A.E., Habermann E.B., Kaplan L.J. (2019). Comfort care in trauma patients without severe head injury: In-hospital complications as a trigger for goals of care discussions. Injury.

